# Vitamin D: Harmless Nutritional Supplement or Serious Medication?

**DOI:** 10.1002/ccr3.70345

**Published:** 2025-03-24

**Authors:** Robert Westhofen, Gerhard Weidinger, Peter Hoffmann, Alexander Daxecker, Lukas Antonitsch

**Affiliations:** ^1^ Department of Medicine, Faculty of Medicine and Dentistry Danube Private University Krems Austria; ^2^ Department for Gastroenterology and Hepatology University Hospital Wiener Neustadt Wiener Neustadt Austria

**Keywords:** acute kidney injury, chronic kidney disease, gastrointestinal bleeding, hypercalcemia, vitamin D supplementation

## Abstract

Excessive vitamin D supplementation may result in significant clinical complications. This case of a 77‐year‐old male demonstrates how unsupervised high doses led to severe hypercalcemia, acute kidney injury, and gastrointestinal bleeding. It underscores the importance of precise dosing, regular monitoring, and medical supervision of vitamin D supplementation.

## Graphical abstract

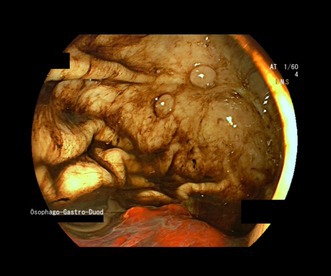

## 1 Introduction

Vitamin D supplementation is beneficial in vitamin D deficiency. However, despite being repeatedly investigated, high dose compared with moderate dose supplementation could not improve bone density [1]. Data on extraskeletal health benefits of vitamin D supplementation beyond correcting vitamin D deficiency by supplements are disappointing [2]. Nonetheless, public interest for vitamin D supplementation significantly rose since the COVID‐19 crisis began [3]. However, over‐the‐counter vitamin D supplements may lead to intoxication, as our case report shows.

Vitamin D deficiency is defined by serum levels below 50 nmol/L. For adults without a deficiency, a daily intake of 800–2000 IU of vitamin D is recommended. In cases of confirmed vitamin D deficiency, higher doses are advised, with 7000–10,000 IU daily or 50,000 IU weekly being appropriate. Regular monitoring of serum levels is essential during supplementation, with an initial reevaluation 8–12 weeks after starting therapy. For those with a deficiency, follow‐up assessments every 3 months are recommended. Daily supplementation exceeding 2000 IU should only be undertaken under medical supervision.

This case report highlights the severe consequences of excessive vitamin D intake in a 77‐year‐old male, illustrating how prolonged high‐dose supplementation led to hypercalcemia, acute kidney injury progressing to chronic kidney disease, and life‐threatening gastrointestinal bleeding requiring extensive transfusion therapy.

## 2 Case Presentation

A 77‐year‐old male patient presented to our clinic's emergency department following a referral from his general practitioner because of dizziness lasting 2 weeks, severe itching in the head and neck area, and significantly elevated creatinine. He denied any relevant preexisting conditions. He appeared well‐nourished and in good general health, with a history of good physical fitness. Apart from taking vitamin D supplements, he reported no regular medication use, and his baseline creatinine level was normal. Especially, no regular consumption of NSAIDs or alcohol was reported.

A more detailed history revealed intake of 80,000–90,000 IU of cholecalciferol daily in liquid form over 6 months, based on advice from an acquaintance about its “bone‐strengthening properties.” The patient did not report any prior bone health‐related issues, such as fractures, family history of fractures, or low bone density.

## 3 Methods (Differential Diagnosis, Investigations, and Treatment)

Physical examination revealed no significant abnormalities, especially no neuropsychiatric or gastrointestinal symptoms.

The results of the laboratory workup as well as reference values are given in Table 1. Laboratory tests indicated acute kidney failure (AKI KDIGO Stage 3) with a creatinine level of 4.6 mg/dL, an eGFR (CKD‐EPI) of 12 mL/min, hypercalcemia with corrected calcium at 3.5 mmol/L, and a normochromic, normocytic anemia with hemoglobin (Hb) of 11.2 g/dL. Parathyroid hormone (PTH) was suppressed at 11.8 pg/mL. Further laboratory tests revealed an increase in free light chains (kappa and lambda), with kappa light chains at 109 mg/L and lambda light chains at 81.1 mg/L. Lactate Dehydrogenase (LDH) levels were within the normal range. Beta‐2‐microglobulin was elevated at 11.4 mg/L. The 25‐hydroxy vitamin D level was above the measurable range, exceeding 600 nmol/L.

**TABLE 1:** Laboratory findings.

Parameter	Initial values	Values at Day 14	Values at Day 30	Values at 20 months post‐discharge	Reference
Creatinine clearance	4.6 mg/dL	5.5 mg/dL	5.5 mg/dL	2.1 mg/dL	0.7–1.2 mg/dL
GFR (CKD‐EPI)	12 mL/min	9 mL/min	9 mL/min	29 mL/min	> 90 mL/min
Corrected calcium	3.5 mmol/L	3.09 mmol/L	2.88 mmol/L	2.2 mmol/L	2.2–2.55 mmol/L
Hemoglobin	11.2 g/dL	5 g/dL	8.5 g/dL	12.6 g/dL	12.5–17.2 g/dL
Parathyroid hormone (PTH)	11.8 pg/mL		10.3 pg/mL		14.9–56.9 pg/mL
ß2 microglobulin	11.4 mg/L				0‐3 mg/L
Kappa light chains	109 mg/L				6.7–22.4 mg/L
Lambda light chains	81.1 mg/L				8.3–27 mg/L
25‐hydroxy vitamin D	> 600 nmol/L		> 600 nmol/L	104 nmol/L	75‐100 nmol/L

The electrocardiogram (ECG) showed no abnormalities, particularly no QTc prolongation.

A venous blood gas analysis showed ionized calcium at 1.82 mmol/L (reference: 1.15–1.27 mmol/L). Other parameters were within normal limits.

A postrenal cause of kidney failure was ruled out via ultrasound in the emergency department.

Chest X‐ray showed no signs of infiltrates or masses.

The patient was admitted to the intermediate care unit. The hypercalcemia was initially treated with forced diuresis (2–3 L of buffered full electrolyte solution in combination with 120 mg of intravenous furosemide per day).

The renal retention parameters initially worsened (Table 1). However, the patient's urine output remained adequate, and there were no criteria for initiating dialysis. Calcium levels gradually decreased; however, on Day 14 after admission, the patient complained of abdominal pain and began passing melena. Norepinephrine was initiated because of hemorrhagic shock. Hb dropped from 10.4 to 5.0 g/dL.

Proton‐Pump Inhibitor (PPI) therapy was initiated (pantoprazole 240 mg/24 h), and the patient received an emergency transfusion of three units of packed red blood cells (PRBC). Gastroscopy revealed erosive gastroduodenitis with hematin streaks throughout the upper gastrointestinal tract. A Forrest III ulcer was seen in the duodenal bulb. Immunohistochemistry was eventually negative for *Helicobacter pylori* .

**FIGURE 1:**
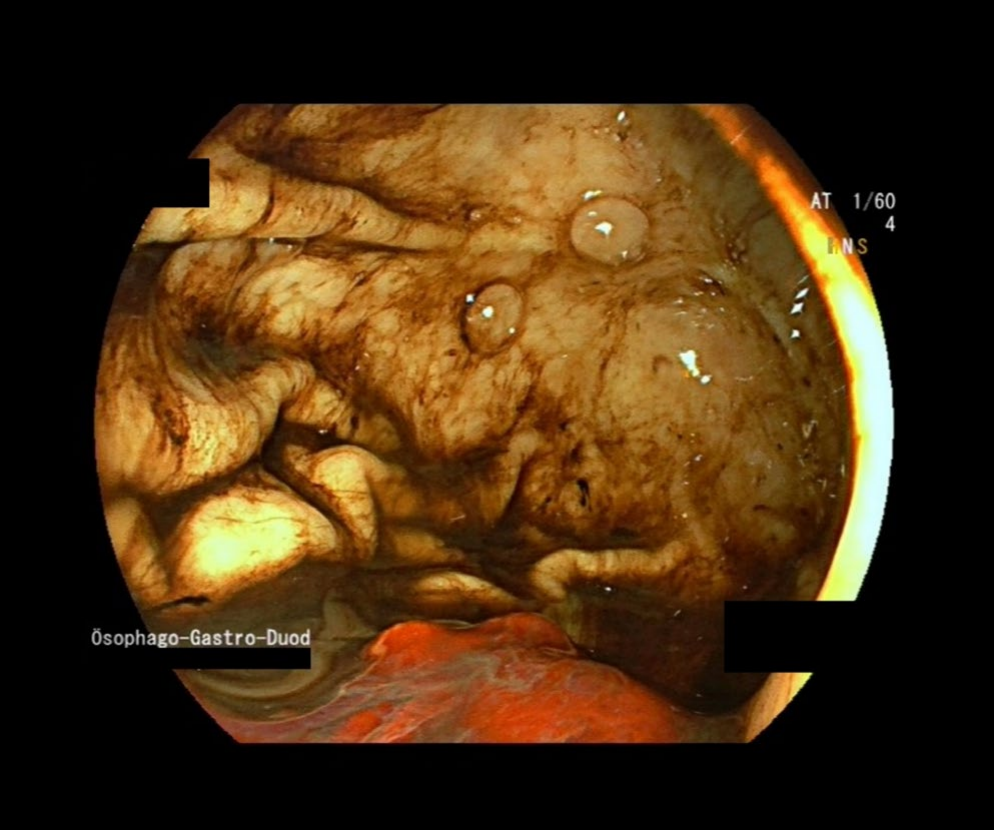
Gastral corpus with hematin and fresh blood.

The patient required ongoing transfusion of several units of PRBC daily. A second gastroscopy on Day 5 revealed a Forrest IIb ulcer in the duodenal bulb, the underlying vessel stump of which was treated with a metal clip and hemostatic agents (Figure 1). The patient's condition stabilized, and catecholamines were gradually tapered. A total of 14 units of PRBC were transfused.

## 4 Conclusion and Results (Outcome and Follow‐Up)

The patient was eventually transferred to a general medical ward. Although forced diuresis reduced calcium levels, significantly elevated renal retention parameters persisted, resulting in chronic kidney disease (CKD) Stage G5, confirmed by a cystatin C eGFR of approximately 9–12 mL/min. Forced diuresis and volume substitution were discontinued. Erythropoietin was administered, leading to a satisfactory increase in hemoglobin levels. Vitamin D supplementation was stopped, and follow‐up at 6 months revealed stable CKD Stage G4 with normal calcium levels. Furthermore, the serum electrophoresis was normal and ß2‐microglobulin levels returned to normal values. The patient's vitamin D levels remained elevated for 20 months after hospital discharge, despite the absence of further supplementation. Six months post‐discharge, the 25‐hydroxycholecalciferol level exceeded 200 ng/mL. At 12 months post‐discharge, it was measurable for the first time at 152 ng/mL. The next available measurement, taken 20 months after discharge, showed a persistently elevated level of 104 ng/mL, still without additional supplementation. The patient's general condition and physical fitness remained good.

## 5 Discussion

We assume that the excessive ingestion of cholecalciferol led to chronic kidney disease Stage G5, which we attribute to long‐standing hypercalcemia, resulting in glomerular [4, 5] and tubular damage [6]. Furthermore, temporary hypoperfusion of the kidneys during hemorrhagic shock may have worsened kidney function.

The transfusion‐dependent upper gastrointestinal bleeding from a vessel stump in the duodenal bulb is likely associated with the existing hypercalcemia. Some studies show increased gastrin secretion in hypercalcemia, potentially leading to ulcers and GI bleeding [6, 7].

Another important differential diagnosis for hypercalcemia in this patient is multiple myeloma (MM). Initial evaluation showed elevated beta‐2‐microglobulin and increased kappa and lambda light chains in the urine. The patient met the CRAB criteria; however, a clear cause for kidney failure, hypercalcemia, and anemia was identified. A follow‐up evaluation for multiple myeloma was negative after 6 months.

Vitamin D toxicity primarily exerts its effects by inducing hypercalcemia. For educational purposes, it is important to note that other symptoms of hypercalcemia include neuropsychiatric disorders, such as depression, anxiety, and cognitive dysfunction. In severe cases, these disturbances can progress to lethargy, stupor, or even coma [8]. Renal complications of hypercalcemia, particularly in cases of prolonged elevation, may include nephrocalcinosis, renal tubular acidosis, and acute or chronic kidney disease, as previously described [5]. Cardiovascular manifestations of hypercalcemia are typically characterized by a shortened QT interval, which can predispose individuals to arrhythmias [9]. Additionally, hypercalcemia can cause various gastrointestinal issues beyond GI bleeding, such as constipation, nausea, and pancreatitis [5, 6].

Taking vitamin D supplements in adequate doses is particularly recommended in Northern Europe, which is commonly practiced, especially in outpatient settings. The use of this supposedly “harmless” supplement is not restricted to pharmacies, as vitamin D capsules with 2000 IU of cholecalciferol per tablet or (as in this case) bottles with 10,000 IU per drop are widely available in drugstores and on popular online marketplaces.

According to the Central European Guidelines [10] from 2017, a daily supplementation of 800–2000 IU is recommended if sufficient sun exposure (45 min daily) is not possible. If serum concentrations fall below 50 nmol/L, a vitamin D deficiency is present, requiring high‐dose supplementation. For adults with a proven deficiency, daily doses of 7000–10,000 IU or weekly doses of 50,000 IU are recommended. After 8–12 weeks, serum levels should be rechecked, with further evaluations every 3 months. For certain risk groups (e.g., pregnant women, dialysis patients, and patients with inflammatory bowel disease), different recommendations apply, and readers are referred to the guidelines for further information.

Despite recommendations for generous use, it is crucial to remember vitamin D's side effects. This case vividly illustrates the potential complications of “blind” and unsupervised use. Therefore, long‐term use and dosage should be discussed with a physician, and regular monitoring of serum levels is recommended for long‐term use, as Paracelsus famously stated: “The dose makes the poison.” This quote aptly summarizes the case described above.

## Author Contributions

**Robert Westhofen:** conceptualization, formal analysis, investigation, methodology, project administration, resources, supervision, validation, visualization, writing – original draft, writing – review and editing. **Alexander Daxecker:** investigation, supervision, validation. **Gerhard Weidinger:** supervision, validation, writing – review and editing. **Peter Hoffmann:** validation, writing – review and editing. **Lukas Antonitsch:** investigation, methodology, supervision, validation, writing – original draft, writing – review and editing.

## Ethics Statement

The ethics and integrity policies were met.

## Consent

Patient consent to publish clinical information and images was obtained in written form using a consent form in German. The patient has agreed to the terms outlined in Wiley's standard consent form, including understanding that their medical information will be published on an open access basis and may be freely accessed worldwide.

## Conflicts of Interest

The authors declare no conflicts of interest.

## Data Availability

All data supporting the findings of this case report are derived from previously published studies, which are cited in the manuscript. The included endoscopy image was obtained with the patient's informed consent for publication, including all accompanying data.
